# Undernutrition during pregnancy in mice leads to dysfunctional cardiac muscle respiration in adult offspring

**DOI:** 10.1042/BSR20150007

**Published:** 2015-06-11

**Authors:** Brittany Beauchamp, A. Brianne Thrush, Jessica Quizi, Ghadi Antoun, Nathan McIntosh, Osama Y. Al-Dirbashi, Mary-Elizabeth Patti, Mary-Ellen Harper

**Affiliations:** * Department of Biochemistry, Microbiology, and Immunology, University of Ottawa, Ottawa, Ontario K1H 8M5, Canada; † Newborn Screening Ontario, Children's Hospital of Eastern Ontario, Ottawa, Ontario K1H 8M8, Canada; ‡ Department of Pediatrics, University of Ottawa, Ottawa, Ontario, K1H 8M5, Canada; § Division of Integrative Physiology and Metabolism, Joslin Diabetes Center, Boston, MA 02215, U.S.A.

**Keywords:** energetics, fetal programming, heart, metabolism, mitochondria, obesity

## Abstract

Intrauterine growth restriction (IUGR) is associated with an increased risk of developing obesity, insulin resistance and cardiovascular disease. However, its effect on energetics in heart remains unknown. In the present study, we examined respiration in cardiac muscle and liver from adult mice that were undernourished *in utero*. We report that *in utero* undernutrition is associated with impaired cardiac muscle energetics, including decreased fatty acid oxidative capacity, decreased maximum oxidative phosphorylation rate and decreased proton leak respiration. No differences in oxidative characteristics were detected in liver. We also measured plasma acylcarnitine levels and found that short-chain acylcarnitines are increased with *in utero* undernutrition. Results reveal the negative impact of suboptimal maternal nutrition on adult offspring cardiac energy metabolism, which may have life-long implications for cardiovascular function and disease risk.

## Abstract (precis)

We show that *in utero* undernutrition is associated with impaired cardiac muscle energetics and increased plasma short-chain acylcarnitines in adult mice. Findings suggest that *in utero* undernutrition is associated with maladaptive programming processes that have negative effects on the heart.

## INTRODUCTION

The developmental programming hypothesis proposes that adverse influences during critical periods in development permanently alter tissue structure and function, which may have persistent consequences for the long-term health of the offspring [1]. Epidemiological studies in humans and animal models have shown that intrauterine growth restriction (IUGR) is associated with an increased risk of developing obesity, insulin resistance and cardiovascular disease in the offspring [2]. IUGR fetuses show a significant decline in cardiac systolic function and children affected by IUGR have been found to have premature stiffening of carotid arteries and elevated blood pressure [3–5]. In rodent models, IUGR offspring are more susceptible to the development of hypertension and cardiac dysfunction in adulthood [6,7]. Although there is a strong association between IUGR and cardiovascular disease, the effect of exposure to maternal undernutrition on energetics in the heart has not been investigated.

Previous research in mice has shown that 50% food restriction during late pregnancy leads to IUGR, low birth weight and offspring that develop progressive, severe glucose intolerance and β-cell dysfunction [8,9]. Using this model, we previously showed that these low birth weight offspring have increased adiposity, decreased skeletal muscle energetics in mixed muscle and a blunted weight loss response to a hypocaloric diet in adulthood [9]. Using adult mice from this subsequent study, we have investigated the effects of *in utero* undernutrition on energetics in the heart and liver. We also report analysis of plasma acylcarnitines, which have previously been shown to be biomarkers of metabolic and/or cardiovascular risk [10–14].

The heart is an organ with a high energy requirement, turning over ~30 kg of ATP daily in humans [15]. Thus, it requires high oxygen uptake to synthesize sufficient ATP by oxidative phosphorylation for proper function. Cardiac diseases are associated with changes in myocardial energy metabolism. A general decrease in oxidative capacity and down-regulation of enzymes of fatty acid oxidation in cardiac muscle have been reported in different models of heart failure [16–21]. Mitochondria in cardiac muscle of individuals with type 2 diabetes mellitus (T2DM) have a decreased capacity for fatty acid-supported respiration [22]. Additionally, in a mouse model of obesity and diabetes, mitochondria have reduced oxidative capacity [23]. We therefore hypothesized that low birth weight induced by maternal undernutrition during late pregnancy leads to long-term impairment of cardiac energy metabolism in offspring.

## MATERIALS AND METHODS

### Animals

All procedures involving the use of animals were performed according to the principles and guidelines of the Canadian Council of Animal Care and the study was approved by the Animal Care Committee of the University of Ottawa. Mice were housed in a facility with controlled temperature, humidity and light-dark cycle (06:00–18:00). Virgin female ICR mice (Harlan; age 6–8 weeks) were paired with male ICR mice (Harlan; age 6–8weeks). Pregnancies were dated by vaginal plug (day 0.5) and pregnant mice were housed individually with *ad libitum* access to standard rodent chow (T.2018; Harlan Teklad). On day 12.5 of pregnancy, dams were randomly assigned to either a control or undernutrition group. In the undernutrition group, food was restricted to 50% that of gestational day matched controls for the remainder of pregnancy. After delivery, mothers were given *ad libitum* access to chow and 24 h after birth, litters were equalized to eight. Pups were weaned at 3 weeks. At 10 weeks, these offspring were randomly assigned to either a 40% calorie restricted group (D01092702: Research Diets) or an *ad libitum* control group. At 14 weeks of age, four groups of mice were studied: *in utero* undernourished offspring fed *ad libitum* postnatally (U-L); *in utero* undernourished offspring that were calorie restricted for 4 weeks during adulthood (U-R); control offspring fed *ad libitum* (C-L); and control offspring that were calorie restricted for 4 weeks during adulthood (C-R). Female mice were used for all determinations, since preliminary work showed a stronger phenotype in female than male offspring. All end-point determinations were performed with mice from at least four different litters per group, with no more than two mice from the same litter.

### Tissue collection

Blood was collected by cardiac puncture. Plasma was stored at −80°C for future analyses. Heart and liver were removed immediately after euthanizing. Myocardial tissue was taken from the left ventricle at the apex of the heart. Approximately 3–5 mg of tissue was used for high-resolution respirometry and the remainder was flash-frozen in liquid N_2_ for Western blot determinations.

### High-resolution respirometry

Tissue (3–5 mg) was homogenized in MIRO5 (0.5 mM EGTA, 3 mM MgCl_2_·6H_2_O, 20 mM taurine, 10 mM KH_2_PO_4_, 20 mM HEPES, 110 mM D-sucrose, 0.1% BSA, 60 mM lactobionic acid; pH 7.1) using the PBI-Shredder SG3 (Oroboros). This mechanical homogenization system applies reproducible force to the tissue yielding standardized preparations. Respiration was determined at 37°C in MIRO5 using the Oxygraph-2k (Oroboros). Malate (2 mM) and octanoyl carnitine (1 mM) were added to determine adenylate-free leak respiration. ADP + Mg^2+^ (5 mM) were subsequently added to determine maximal electron flow through electron-transferring flavoprotein and fatty acid oxidative capacity. Glutamate (10 mM) and succinate (10 mM) were then added to determine maximum oxidative phosphorylation capacity. Oligomycin (2 μg/ml) was added to determine oligomycin-induced leak respiration. Antimycin A (2.5 μM) was added to inhibit complex III and terminate respiration to determine non-mitochondrial oxygen consumption. All values were corrected for residual oxygen consumption. The oxygen consumption [pmol/(s·mg)] was expressed relative to citrate synthase activity (μmol/min/mg).

**Table 1:** Mouse characteristics at age 14 weeks C, control offspring; U, *in utero* undernourished offspring; L, fed *ad libitum*; R, after a 4 week 40% calorie restriction. Values are mean ± S.E.M., *n*–8–9. Two-way ANOVA with Bonferroni post-hoc test.

	C-L	C-R	U-L	U-R
Body mass (g)	31.96±1.34*	24.78±0.53†	33.81±2.61‡	28.03±0.65
Tail length (cm)	9.3±0.1	9.3±0.1	9.4±0.1	9.2±0.1
Femur length (cm)	1.7±0.1	1.7±0.1	1.7±0.1	1.7±0.1
Heart (g)	0.138±0.005	0.123±0.003	0.123±0.007	0.120±0.004
Liver (g)	1.235±0.042	1.307±0.049	1.164±0.091	1.219±0.039
gWAT (g)	0.572±0.068§	0.375±0.019a	1.169±0.285	0.708±0.053

*P<0.01 (L compared with R).

†*P*<0.1 (C compared with U).

‡*P*<0.05 (L compared with R).

§*P*<0.05 (C compared with U).

### Citrate synthase activity

Citrate synthase activity was determined in homogenized tissue according to the method of Srere [24].

### Western blotting and sample preparation

Left ventricular cardiac muscle was homogenized on ice in a lysis buffer (20 mM Tris/HCl, 1% Triton X-100, 50 mM NaCl, 250 mM sucrose, 2% β-mercaptoethanol, 50 mM NaF, 5 mM NaPP, 1 mM Na_3_VO_4_ and protease inhibitors) and spun at 14000 ***g*** for 20 min at 4°C. Protein content was measured using a bicinchoninic acid assay and samples were stored at −80°C. Samples were subjected to reducing SDS/PAGE. Proteins were electroblotted on to nitrocellulose membranes and stained with Ponceau S. After blocking for 1 h at room temperature in 5% skim milk, incubation in primary antibody was overnight at 4°C. The following primary antibodies were used at the indicated dilutions: ANT (adenine nucleotide translocase; Santa Cruz Biotechnology; 1:1000), UCP3 (uncoupling protein 3; Abcam; 1:1000), MitoProfile Total OXPHOS Rodent WB Antibody Cocktail (Abcam; 1:800), vinculin (Abcam; 1:10000). Following 3 × 10 min washes with TBS+0.1% Tween-20, membranes were incubated in the appropriate horseradish peroxidise-conjugated secondary antibody diluted in 5% skim milk at room temperature for 1 h. Bands were visualized using enhanced chemiluminescence. Band intensity was quantified by density analysis using ImageJ (NIH) and normalized to vinculin.

### Plasma acylcarnitine measurement

A 3.2 mm punch from a dried plasma sample collected on Whatman 903™ filter paper card was placed into the designated well of a 96-well plate. Samples were collected from 14 week old mice. Samples were prepared at the Newborn Screening Ontario laboratory based on methods described by Turgeon et al. [25] with minor modification. Briefly, target analytes were extracted from the dried plasma samples using a methanolic solution containing isotope-labelled internal standards. Samples were evaporated under nitrogen followed by derivatization using butanolic-HCl at 60 °C for 20 min. Excess reagent was evaporated to dryness using nitrogen and the residue was reconstituted with 80% acetonitrile solution. A 10 μl portion of sample was injected into an MS/MS system consisting of a Waters TQ Detector, Waters 1525μ Binary HPLC Pump and a Waters 2777C Sample Manager (Waters). MS/MS analysis of target analytes was achieved using a combination of multiple reaction monitoring and precursor ion scans, with the ESI source being operated in the positive ion mode.

### Statistical analyses

All measures were analysed using GraphPad Prism, version 5.0. Differences between groups were analysed using a Student's t-test or two-way ANOVA followed by Bonferroni post-hoc tests, as appropriate. Values are reported as mean ± S.E.M. *P*<0.05 was considered significant.

## RESULTS

Body weight 24 h after birth was 26% reduced in U compared with C (Supplementary Figure S1). As expected, based on previously published work, there was no difference in linear growth as assessed by tail and femur length at age 14 weeks (Table 1). Body weight between C-L and U-L was not different (Table 1). This indicates that U experience catch-up growth, which has been shown to be required for the development of the phenotype in this model [26]. Additionally, there was no difference in the weight of the heart or liver (Table 1). U had increased gonadal white adipose tissue (gWAT; Table 1).

### *In utero* undernutrition decreases energetics in heart homogenate

Respiration in left ventricular cardiac muscle homogenate from 14-week-old mice was assessed under different conditions using high-resolution respirometry and normalized to citrate synthase activity, a marker of mitochondrial content. Adenylate-free leak respiration (Figure 1A), fatty acid oxidative capacity (Figure 1B), maximum oxidative phosphorylation capacity (Figure 1C) and oligomycin-induced leak respiration (Figure 1D) were reduced in U compared with C. Calorie restriction also decreased adenylate-free leak respiration, fatty acid oxidative capacity and maximum oxidative phosphorylation capacity (Figure 1). Similar differences between U and C were also observed at 10 weeks of age (Supplementary Figure S2). There was no difference in citrate synthase activity between groups.

**Figure 1:**
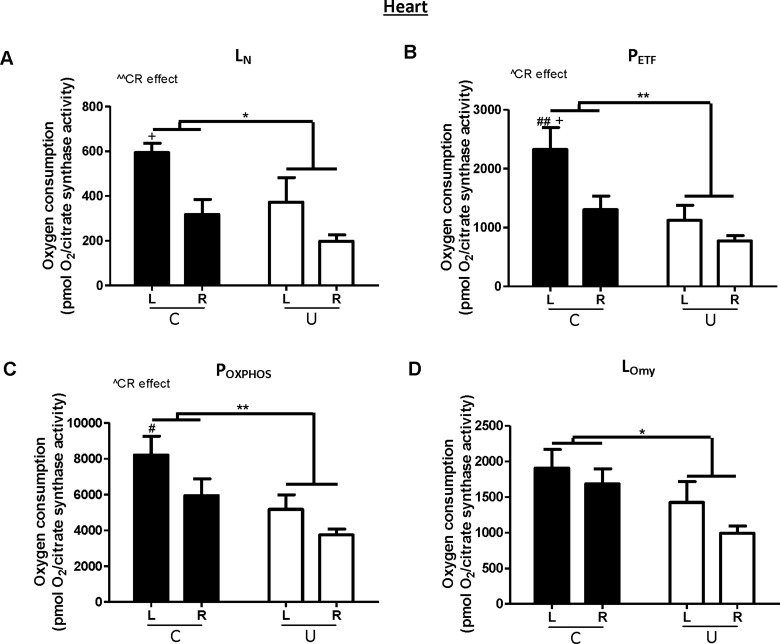
*In utero* undernutrition alters energetics in heart homogenate O_2_ flux in heart homogenate from 14–week-old mice fed *ad libitum* (*L*) and after a 4 week 40% calorie restriction (*R*). Values are expressed relative to citrate synthase activity. Data are shown for adenylate free leak respiration (*L*_N_; **A**), maximal electron flow through electron-transferring flavoprotein and fatty acid oxidative capacity (*P*_ETF_; **B**), maximum oxidative phosphorylation capacity (*P*_OXPHOS_; **C**) and oligomycin-induced leak respiration (*L*_omy_; **D**). Values are mean ± S.E.M., *n*=6–8, *P<0.05, **P<0.01, #*P*<0.05 (C compared with U), ##*P*<0.01 (C compared with U), +*P*<0.05 (L compared with R), ^∧^*P*<0.05 [calorie restriction (CR) effect], ^∧∧^*P*<0.01 (CR effect). Black=C (control offspring), white=U (*in utero* undernourished offspring).

### *In utero* undernutrition does not alter energetics in liver homogenate

In contrast with the effects of *in utero* undernutrition on energetics in heart, adenylate-free leak respiration (Figure 2A), fatty acid oxidative capacity (Figure 2B), maximum oxidative phosphorylation capacity (Figure 2C) and oligomycin-induced leak respiration (Figure 2D) did not differ between U and C in liver homogenate. Calorie restriction also did not have a significant effect in the liver (Figure 2). Citrate synthase activity in liver was not different between the groups. Therefore, under the conditions assessed there were no effects on energetics in liver tissue.

**Figure 2:**
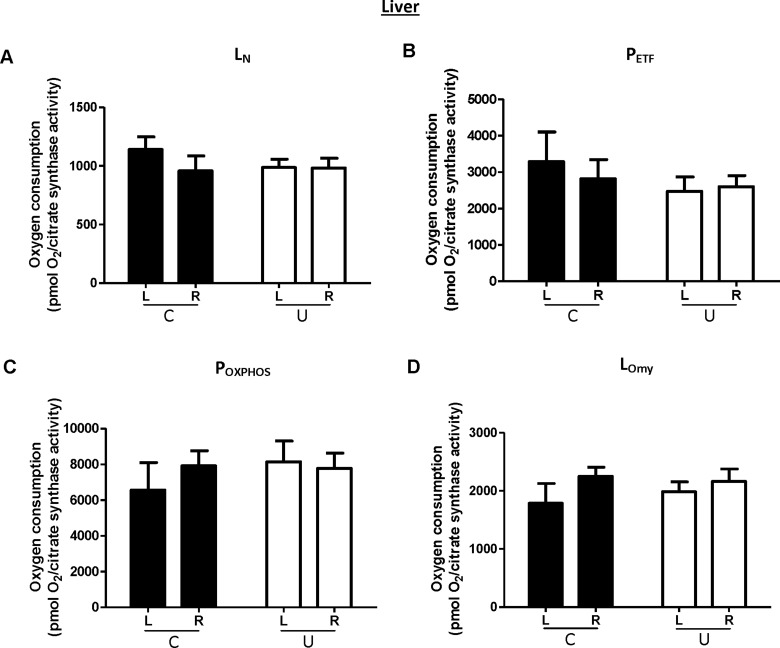
*In utero* undernutrition does not alter energetics in liver homogenate O_2_ flux in liver homogenate from 14-week-old mice fed *ad libitum* (*L*) and after a 4 week 40% calorie restriction (*R*). Values are expressed relative to citrate synthase activity. Data are shown for adenylate free leak respiration (*L*_N_; **A**), maximal electron flow through electron-transferring flavoprotein and fatty acid oxidative capacity (*P*_ETF_; **B**), maximum oxidative phosphorylation capacity (*P*_OXPHOS_; **C**) and oligomycin-induced leak respiration (*L*_omy_; **D**). Values are mean ± S.E.M., *n*=6–8. Black=C (control offspring), white=U (*in utero* undernourished offspring).

### *In utero* undernutrition does not alter mitochondrial content in the heart

Although respiration values were normalized to citrate synthase activity, the decreased heart energetics in U could be due in part to differences in the amount of one or more of the mitochondrial complexes. Therefore, we assessed protein levels of mitochondrial complexes I–V. Protein expression of representative proteins for the five complexes did not differ between U and C (Figure 3). Given the observed difference in leak-dependent respiration, we also measured protein levels of ANT (Figure 4A) and UCP3 (Figure 4B). There were no differences in the expression of these proteins.

**Figure 3:**
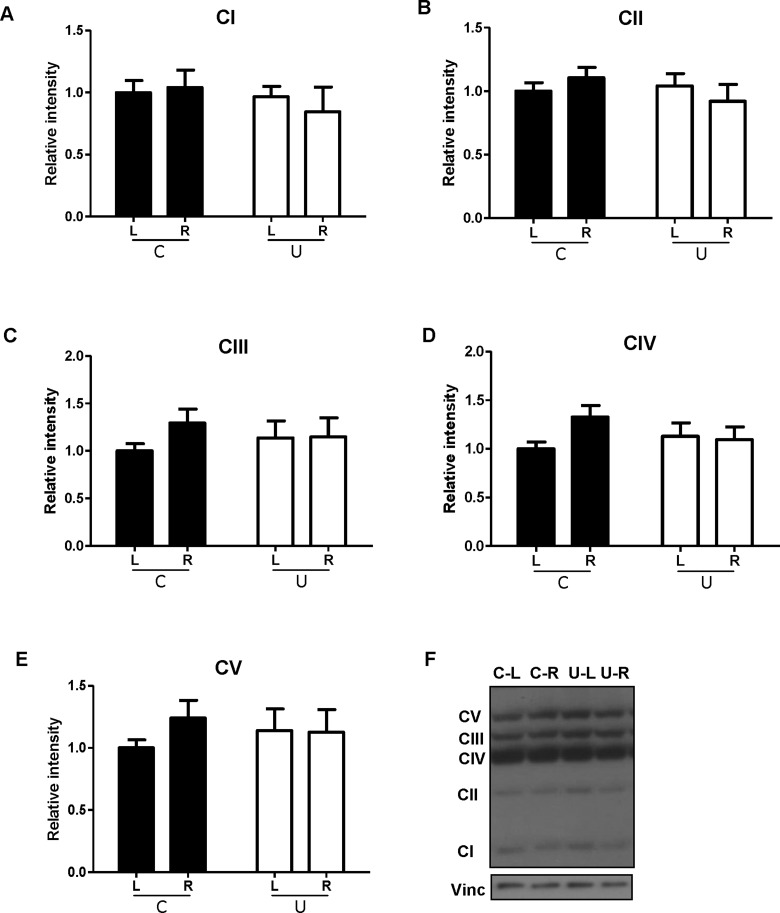
*In utero* undernutrition does not alter mitochondrial content in the heart Protein expression of mitochondrial complexes normalized to vinculin (Vinc) loading control in heart homogenate from 14-week-old mice fed *ad libitum* (*L*) and after a 4 week 40% calorie restriction (*R*). Quantification of complex I (CI; **A**), II (CII; **B**), III (CIII; **C**), IV (CIV; **D**) and V (CV; **E**) expression and representative Western blot (**F**). Values are mean ± S.E.M., *n* = 8. Black=C (control offspring), white=U (*in utero* undernourished offspring).

**Figure 4:**
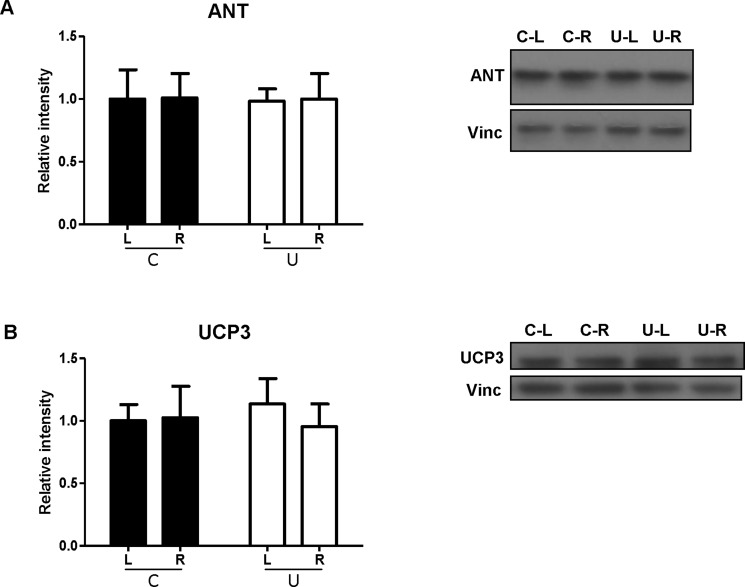
*In utero* undernutrition does not alter expression of ANT or UCP3 Protein expression of ANT (**A**) and UCP3 (**B**) normalized to vinculin (Vinc) loading control in heart homogenate from 14–week-old mice fed *ad libitum* (*L*) and after a 4 week 40% calorie restriction (*R*). Quantification, left and representative Western blot, right. Values are mean ± S.E.M., *n*=8. Black=C (control offspring), white=U (*in utero* undernourished offspring).

### Plasma short-chain acylcarnitines are increased with *in utero* undernutrition

Impairments in fatty acid oxidation can result in the accumulation of intermediary metabolites, such as acylcarnitines. Given the current finding of decreased fatty acid oxidation in cardiac muscle and our previous finding of decreased fatty acid oxidation in skeletal muscle [9], we measured plasma acylcarnitines using MS/MS. Under *ad libitum* fed conditions, U had increased acetylcarnitine (C2; Figure 5A) and short-chain acylcarnitines (C3–C5; Figure 5B) compared with *ad libitum* fed controls (U-L compared with C-L). There was no difference in medium- (C6–C12; Figure 5C) or long-chain (>C12; Figure 5D) acylcarnitines between U-L and C-L. Interestingly, in U, calorie restriction significantly decreased acetylcarnitine, short-, medium- and long-chain acylcarnitines (U-L compared with U-R; Figures 5A–5D). In C, there was no significant effect of calorie restriction on acylcarnitine levels. However, there was a trend for decreased acetylcarnitine (C-L compared with C-R; Figure 5). Complete results of the plasma acylcarnitine analysis are provided in Supplementary Table S1.

**Figure 5:**
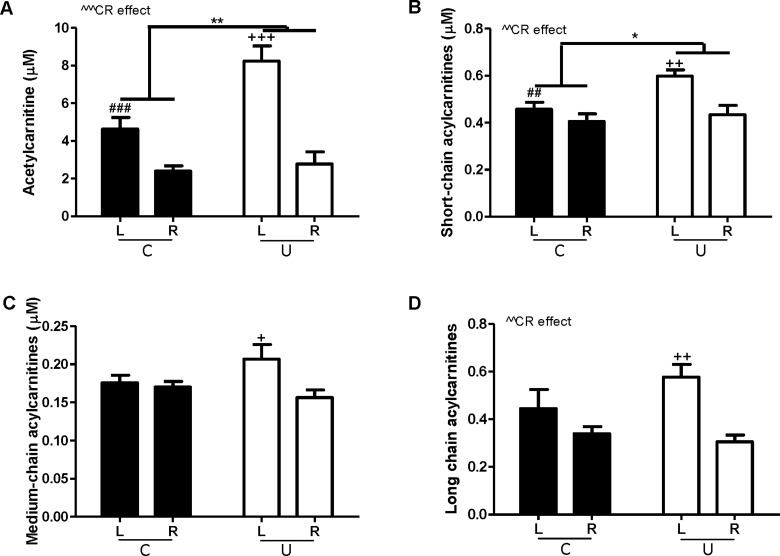
Plasma short-chain acylcarnitines are increased with *in utero* undernutrition Acylcarnitines measured in plasma from 14-week-old mice fed *ad libitum* (*L*) and after a 4 week 40% calorie restriction (*R*). (**A**) Acetylcarnitine; (**B**) sum of short-chain acylcarnitines (C3–C5); (**C**) sum of medium-chain acylcarnitines (C6–C12); (**D**) sum of long-chain acylcarnitines (>C12). Values are mean ± S.E.M., *n*=7–8, *P<0.05, **P<0.01, ##*P*<0.01 (C compared with U), ###*P*<0.001 (C compared with U), +*P*<0.05 (L compared with R), ++*P*<0.01 (L compared with R), +++*P*<0.001 (L compared with R). Black=C (control offspring), white=U (*in utero* undernourished offspring).

## DISCUSSION

In the present study, we show that *in utero* undernutrition in mice results in impaired cardiac muscle energetics without altering mitochondrial content and that this is associated with increased plasma short-chain acylcarnitines. Although many studies have shown an association between IUGR and cardiovascular disease, the effects of *in utero* undernutrition on heart energetics have not been characterized. Given the strong association between IUGR and cardiovascular disease, our data demonstrate that maternal food restriction alters metabolism in the heart, which influences the cardiovascular health and disease risk in offspring.

Mitochondrial respiration in heart homogenate was decreased in U compared with C. Protein expression of mitochondrial complexes I–V in the heart was not different between U and C, indicating that the reduced respiration was not due to a decrease in mitochondrial content or the expression of one of the respiratory chain complexes. The observed differences in respiration may be due to differences in the activity of one or more of these complexes. Proton leak requires the activation of mitochondrial anion carrier protein function, which can be catalysed by ANT and UCP3 [27–29]. The decrease in proton leak respiration measured in U does not appear to be due to differences in protein expression of ANT or UCP3 but may be due to differences in their activity or post-translational modification. Therefore, future research should investigate possible changes in the composition of mitochondrial super-complexes and in protein post-translational modifications (e.g., phosphorylation and/or glutathionylation of respiratory chain and other mitochondrial proteins). We also cannot exclude the possibility that differences are in part due to structural changes in the mitochondria that may make them more susceptible to damage during sample preparation.

Our finding of decreased respiration in cardiac tissue in this mouse model is consistent with the altered energetics reported in heart tissue of other rodent models of diabetes and in patients with diabetes. It has been shown that mitochondria from diabetic human heart have an impaired capacity to oxidize palmitoyl-carnitine [22] and in a mouse model of obesity and type 2 diabetes mitochondria were shown to have a decreased oxidative capacity [23]. Furthermore, there is a decrease in the expression of fatty acid oxidation genes and enzymes and a decrease in myocardial energy production in heart failure patients and in animal models of heart failure [16–18,20,21]. Therefore, our results suggest that the decreased oxidative capacity in the heart of *in utero* undernourished offspring may contribute to their increased risk of cardiovascular and other metabolic diseases.

We have shown that respiration in heart tissue is decreased at 10 weeks of age and remains decreased at 14 weeks of age; however, future work is needed to determine the time course of these metabolic derangements. Studies have shown that IUGR fetuses and neonates show changes in cardiac morphology and function [3,4,30–32], which, combined with the results presented here, suggest that IUGR may be associated with cardiac metabolic adaptations *in utero* that become detrimental in later life. The present study was limited to the left ventricle. This region of the heart was chosen due to its relatively high rate of oxygen consumption (approximately twice that of the right ventricle) and because the left ventricle has been most frequently studied in clinical and animal models [33]. Thus, it remains to be determined whether there is impaired energetics in other regions of the heart.

We also studied energetics in liver tissue but did not detect any differences between U and C under the conditions tested. Previously, in the same model, IUGR had no effect on hepatic insulin resistance [8]. However, our finding was surprising given that others have found decreased mitochondrial DNA content and decreased oxygen consumption in isolated mitochondria from the liver of IUGR rats and piglets [34–36]. This discrepancy may be due to differences in the animal and model of IUGR used. Previous studies were also limited to the use of isolated mitochondria. The *in situ* approach we have used allows the analysis of mitochondrial function within an integrated cellular system, preserving interactions with other cell components that are important for metabolic channelling and intracellular energy transfer [37–39]. We also cannot exclude the possibility that effects in the liver may not be observed until later in life.

Our findings demonstrate that calorie restriction had a significant effect on cardiac muscle energetics, decreasing adenylate free leak respiration, fatty acid oxidative capacity and maximum oxidative phosphorylation capacity. Cardiac respiration was also reduced in U, demonstrating that the heart is a tissue that responds to negative energy balance at the whole body level. Interestingly, we observed a significant decrease in adenylate free leak respiration and fatty acid oxidative capacity with calorie restriction in C (C-L compared with C-R), but not in U (although there appears to be a similar trend).

Overall, we have shown that U has decreased fatty acid-supported respiration in left ventricular cardiac muscle. Previously, we found that fatty acid-supported respiration was decreased in permeabilized muscle fibres from white gastrocnemius of U mice [9]. Interestingly, in individuals with T2DM high-energy phosphate metabolism is impaired in both cardiac and skeletal muscle. In cardiac muscle, individuals with T2DM have a decreased phosphocreatine (PCr)–ATP ratio and in skeletal muscle, PCr loss was faster during exercise and PCr recovery was slower after exercise [40]. Our findings in heart and muscle suggest that U have an impaired ability for fatty acid oxidation, which may be linked to an increase in incomplete fatty acid oxidation. Consistent with this hypothesis, we found that short-chain acylcarnitines are increased in *ad libitum* fed U compared with *ad libitum* fed C. Calorie restriction decreased short-chain acylcarnitines in U but not in C, and normalized acylcarnitine levels to that of the calorie restricted controls. Interestingly, higher levels of short-chain acylcarnitines have been reported in patients with T2DM and in patients with metabolic syndrome [12,41]. It has been suggested that insulin resistance may be linked to incomplete fatty acid β oxidation and the resulting increase in acylcarnitines [42]. In primary myotubes, the reduction of short-chain acylcarnitine and acetylcarnitine formation was shown to protect against palmitate-induced insulin resistance [42]. Taken together, we suggest that the decrease in fatty acid-supported respiration seen in U may result in an increase in incomplete fatty acid oxidation and the subsequent increase in short-chain acylcarnitines, which may be linked to the development of insulin resistance in this model. This idea will be explored further in future research.

In summary, we have shown that *in utero* undernutrition results in dysfunctional cardiac muscle energetics. Furthermore, *in utero* undernutrition increases plasma short-chain acylcarnitines. These findings support the hypothesis that *in utero* undernutrition is associated with a maladaptive programming process that has negative effects on the heart. A better understanding of mechanisms mediating the cardiovascular effects of maternal nutrition on adult offspring should aid in the design of interventions aimed to prevent these detrimental effects and improve health. We conclude that *in utero* undernutrition in late pregnancy alters energetics in the heart, which could have lifelong implications for cardiovascular function.

## Abbreviations

ANT: adenine nucleotide translocase
gWAT: gonadal white adipose tissue
IUGR: intrauterine growth restriction
PCr: phosphocreatine
T2DM: type 2 diabetes mellitus
UCP3: uncoupling protein 3
